# Military nurses’ Experiences of Interprofessional education in Crisis Management: a Qualitative Content Analysis

**DOI:** 10.30476/jamp.2021.87653.1317

**Published:** 2021-04

**Authors:** ZOHREH VAFADAR, MOHAMMAD HOSSEIN AGHAEI, ABBAS EBADI

**Affiliations:** 1 Health Management Research Center, Baqiyatallah University of Medical Sciences, Tehran, Iran; 2 Nursing Faculty, Baqiyatallah University of Medical Sciences, Tehran, Iran; 3 Clinical Research Center of Baqiyatallah Hospital, Military Nursing Department, Nursing Faculty, Baqiyatallah University of Medical Sciences, Tehran, Iran; 4 Behavioral Sciences Research Center, Life style institute, Baqiyatallah University of Medical Sciences, Tehran, Iran

**Keywords:** Management, Interprofessional education, Nurses, Qualitative analysis

## Abstract

**Introduction:**

Today’s human health care needs are so complex and dealing with them requires interprofessional collaboration and teamwork, which must be learned by the appropriate educational methods. In this regard, the Crisis Management Interprofessional Education program (CMIPE) in the form of desktop maneuver with the presence of the military nurses and other professional groups involved in the crisis was designed and implemented in Military Nursing School in Tehran, Iran. The aim of this study was to explore the experiences of the military nurses of participating in an interprofessional education program in crisis management domain.

**Methods:**

This was an exploratory case study using conventional content analysis of the experiences of 28 military nurses participating in this program. The qualitative data were collected with three methods including observation in the field (note-taking and filming) and an open-ended questionnaire and two focus group discussions with military nurses.

**Results:**

Qualitative analysis of the experiences of military nurses led to the emergence of four overarching themes which included professional mutual recognition, shared mental models, valuing joint responsibility and collaboration, perceived self-worth as a member of an interprofessional team.

**Conclusion:**

The crisis management education based on an interprofessional approach created new experiences for military nurses by bringing together professional groups involved in a crisis in a constructive interaction, and with emphasis on learning from each other. This empowers them to provide teamwork and interprofessional collaboration in a critical situation, and therefore, enhancing their ability to cope with different crises.

## Introduction

Today’s human health care challenges and needs are so complex that they cannot be met by one specialty or a particular professional group alone. Therefore, a team of different health professions with effective and constructive communication is required to meet the increasing needs of humans in a complex environment affected by various elements. The public health challenges faced by the new world, such as epidemiological transition, the emergence of pandemics such as Ebola and Covid-19, along with inherited challenges such as aging, growing burden of non-communicable diseases, medical errors, and shortage of skilled health professionals, have left no options except for interprofessional collaborations and teamwork. In other words, dealing with the complicated health care needs interprofessional collaboration and teamwork competencies, which must be learned by the appropriate educational methods and careful planning ( 1 ). There is increasing evidence to suggest that lack of effective teamwork in health care systems has led to reduced quality and security of care, failure to understand patients’ problems, discontinuous and fragmented care, increasing of conflict among different health professions, widespread preventable medical errors, increasing cost of ineffective treatments, and lack of people’s equitable access to health services ( 1 , 2 ).

 In addition to the challenges of meeting the complex health care needs, regarding the spread of crises internationally, the readiness of various health professions to deal with the crisis has become an urgent need. Dealing with a crisis is a great team effort, thus professions being at the forefront crisis response must have teamwork competencies ( 3 ). These competencies include effective communication, professional mutual recognition and trust, information sharing, common goal setting and decision making, group cohesion, shared collective responsibility, conflict resolution, problem-solving, ethical sensitivity, and leadership ability. Acquiring these competencies requires appropriate educational methods ( 4 , 5 ). Health professions are at the forefront of responding to natural and man-made crises with human casualties, hence their preparedness and effective responses are the important factors in determining the number of deaths and possible injuries in critical events ( 6 , 7 ). Nurses in general and military nurses in particular, as the largest health professional group in critical situations, are involved in an unpredictable situation with limited resources for care and at the same time experience a high level of psychological stress. If they are not prepared in advance for crises, it will have adverse health consequences ( 8 , 9 ). 

Unfortunately, the experiences of recent crises show that the nurses are not only unprepared to respond to the crisis but also inconsistencies and conflicting actions between professional groups involved in the crisis worsen the situation and increase casualties ( 10 , 11 ). After September 11, and following terrorist attacks on the World Trade Center in New York, educational planning to improve nurses’ readiness increased from 32.7% in 2001 to 53% in 2003 ( 12 ). In designing the nurses' curricula for critical situations, the World Health Organization emphasizes the acquisition of important competencies such as effective communication, the leadership of interprofessional teams, problem-solving and joint decision-making, critical thinking and conflict resolution, and the safety of individuals and families by providing evidence-based care ( 13 ). 

 Willems, et al. reviewed the non-technical skills required to respond to crises by health professions. These skills include effective interpersonal communications, the ability to lead teams, resolving conflict, flexibility, responsibility, psychological and physical self-care, collaboration, and coordination. They concluded that these capabilities would not be achievable unless individuals were trained in a network of interprofessional interactions ( 14 ). Josed, et al. by reviewing more than 190 initial studies showed that interprofessional education (IPE) and simulation were the most effective educational methods to improve the ability of nursing students to respond to a crisis ( 15 ). Hillier, et al. in a systematic review study showed that IPE is the most effective model for promoting teamwork and inter-organizational collaboration in health care systems ( 16 ). Mackintosh and his colleagues showed in their study that IPE is a good tool to develop teamwork ability in health science students ( 17 ).

The World Health Organization (1973) has warned about lack of teamwork abilities in health care system graduates to meet the health needs of communities. In 1988, the WHO proposed InterProfessional Education (IPE) as an effective strategy to achieve teamwork care and enhance patients’ health outcomes. IPE has now proved itself as an effective strategy to improve safety and quality of care worldwide ( 18 , 19 ). According to WHO, IPE occurs when two or more students in the fields of health science are educated at the same time, learn together, and from and about each other, in order to provide collaborative and patient-centered care in an effective manner ( 20 ). During the past decades, research into the development of interprofessional education in the world has increased dramatically ( 1 ). 

Iran with a population over 80 million, located in Central Eurasia, is one of the ten most disaster-prone countries in the world. It is the sixth country in the world and the fourth country in Asia in terms of the occurrence of accidents and disasters. Almost 90% of its population is exposed to natural disasters. Out of 40 natural disasters in the world, 31 have occurred in Iran ( 7 , 21 , 22 ). Unfortunately, there is some evidence that nurses' preparedness to deal with crises is poor ( 6 , 23 ). Weakness in the teamwork of health profession groups is one of the important causes of increasing human casualties and wasting time, energy, and financial resources in critical events in Iran ( 22 , 24 , 25 ). The experiences of health managers in providing relief in the Bam, Kerman earthquake (December  26, 2003) with 50000 dead, and also floods in April 2019 in the western and southwestern regions of Iran showed that weakness in information exchange, poor coordination, and collaboration, ambiguity in the roles and conflict between different medical and relief teams, multiple decision-making centers and weakness in joint decision-making, the lack of a common language among relief teams as well as inconsistencies between organizations responsible for the crisis have been the most challenges facing relief in these great disasters, leading to an increase in casualties. One of the major challenges in our context is the lack of familiarity of organizations involved in crisis management with the teamwork ( 3 , 26 ). These conditions indicate a gap in appropriate educational methods and health professions preparation for critical situations ( 6 , 27 ). Experiences have shown that even a small investment in coordination and teamwork, especially between military and other organizations leads to better outcomes in crisis management ( 28 ). Achieving coordination and teamwork between the various professional groups involved in the crisis requires an appropriate platform; one of the important platforms is interprofessional education and simulated maneuvers ( 6 ). In Iran, uniprofessional education is a predominant educational method to prepare health professional groups in the face of crisis. Different health professions are taught in separate environments without the opportunity to interact and share information, recognize each other’s roles and common goal setting. This type of education not only fails to prepare them for teamwork, but also creates an individualistic spirit in them ( 29 ).

Unfortunately, despite emphasizing the importance of IPE in developing the competencies needed to deal with the crisis in military nurses, fewer steps have been taken to develop it; at the same time, little research has been performed in this field in Iran. Therefore, there is still no accurate knowledge of its implementation in our health education system ( 29 , 30 ). 

Military nurses will face foreseen and unforeseen crises in the coming years; hence, their ability to respond to these crises is the most important determinant of health, quality of life, and sustainable development of society. They should, therefore, be prepared to deal with crises by using the new educational methods ( 31 ). 

In this regard, the Crisis Management Interprofessional Education program (CMIPE) in the form of desktop maneuver was designed and implemented with three main objectives: 1- introducing an effective method of interprofessional crisis management to our context 2- improving the capability of military nurses in dealing with crisis 3- exploring the military nurses' experiences of participating in crisis management education in the form of interprofessional desktop maneuver. In this article, we address the third objective and explain the military nurses' experiences of participating in this program. 

## Methods

This was an exploratory case study utilizing conventional content analysis of experiences of military nurses participating in the interprofessional crisis management program in May 2018 in Military Nursing School in Tehran, Iran.

### Intervention 

A desktop maneuver is an innovative method to increase the ability of nurses in the face of crises ( 23 ). First, by announcing, nurses working in three military hospitals in Tehran were invited to participate in the Crisis Management Interprofessional Education program (CMIPE). Twenty eight military nurses participated in this program. Military nurses’ mean age was 34.8 years with a standard deviation of 6.9 and work experience with an average of 11.7 years and a standard deviation of 6.6. 55% were men, 88.3% had a bachelor's degree, 11.7% had a master's degree, and 51.7% had a history of being in a real critical situation. Eight military doctors, eight firefighters, and eight Red Crescent members (all male) were invited to form interprofessional teams with military nurses by the dean of the Military Nursing School. Each team consisted of at least four military nurses, one military doctor, one firefighter, and one Red Crescent member. The Crisis Management Interprofessional Education program was implemented for two days from 8 am to 5 pm, being conducted based on the Hospital Incident Command System. On the first day, interprofessional teams were formed. Initially, crisis management was presented theoretically based on Hospital Incident Command System, and after that the desktop maneuver was performed by presenting crisis situations scenarios to interprofessional teams. Each interprofessional team had to analyze critical situations scenarios and present appropriate plans or solutions to deal with the crisis in scenarios through discussion, information sharing, and decision-making by all team members with a final report. At the end of the maneuver, representative of each team submitted their final report to the other teams and the reports were reviewed and criticized by the other teams, and the final summary for each scenario was presented and recorded. Educational content and critical situation scenarios were designed and validated by a panel of experts consisting of seven professors with specialties in health management in disasters, medical education, crisis preparedness and interprofessional education.

### Data collection

 The qualitative data were collected with three methods, including observation in the field (note-taking and filming), open-ended questionnaire and focus group discussion with military nurses. One of the members of the research team (Z,V), as an observer participant, in all stages of the desktop maneuver, recorded and filmed each team discussion and the performances; she also filmed the oral reports of representatives of the teams. At the end of the workshop, a questionnaire with an open-ended question was given to all military nurses and they were requested to write their experiences from participating in CMIPE program. In addition, at the end of the workshop, the volunteer nurses were invited to participate in two focus group discussions to review their experiences, with the main questions of what are your experiences of attending the Crisis Management Interprofessional Education program؟ What happened to you in this program? Focus group is a popular method of collecting qualitative data and its key feature is the interaction between the participants and the researcher, synergistically providing a dynamic and appropriate environment to discuss perceptions, experiences, thoughts, and ideas of the participants. By sharing experiences between groups’ members, a deep understanding of the phenomenon under study is developed. Four military nurses attended each focus group; both focus group sessions were held at the Nursing School, and each session lasted almost 45 to 60 minutes. 

### Ethical considerations

All steps of data collection were performed with informed consent and the ethical principles of confidentiality and anonymity. The permission and moral approval to conduct this study was obtained from the Clinical Research Center of military Hospital and the Research Ethics Committee of Military University of Medical Sciences in Tehran, Iran, with the ethical code IR.Bmsu.Rec.1396.143. In all stages of the study, confidentiality of information was considered, and the samples were free to be excluded from the study at any time.

### Data analysis

 Of the 28 participating military nurses, 18 presented their experiences in the open-ended questionnaire. The research team viewed videos taken from the activities of interprofessional teams and recorded commentaries along with field notes. The focus group discussion was recorded and transcribed word by word. To analyze the data, the conventional content analysis method introduced by Hsieh &amp; Shannon was used ( 32 ). Its steps include 1-Aggregating the textual data of the open-ended questionnaire, field notes and focus group discussion, 2-Repeated reading of the whole texts to gain a general understanding of them 3-Explaining semantic units based on the purpose of the study, 4-Coding of explained semantic units, 5-Frequent and continuous comparisons of codes and classifying them into different classes based on their similarities and differences, 6-Comparison of classes to determine the final themes 7-Explaining the main themes covering all other classes and expressing the concepts hidden in the participants' experiences. MAXDA10 software (version, 2010) was used to manage qualitative data in the content analysis process. 

### Rigor

To confirm the trustworthiness of this study, the validity criteria for qualitative research were employed, including acceptability, reliability, conformability and transferability ( 33 ). Acceptance or credibility was achieved through the researcher's constant engagement with the data and full presence in the field. Data triangulation was accomplished using an open-ended questionnaire, group discussions, observations, and field notes as well as the filming of team performances. The results were announced to the participants and their approval or disapproval regarding the consistency of the results with their opinions being examined. The steps of data analysis were accomplished by two members of the research team (Z, V and A, E) separately and compared; meanwhile, the data collection and analysis processes were reviewed by one of the professors in the expert panel. Transferability can also be achieved by fully explaining all steps and extracting as well as analyzing the data and coding and determining the classes.

## Results

The qualitative analysis of the experiences of participating military nurses in CMIPE led to the emergence of four overarching themes, including professional mutual recognition, shared mental models, valuing joint responsibility and collaboration, and perceived self-worth as a member of an interprofessional team.

In our context, IPE is less known in practice and therefore not commonly used, thus it was a new experience for the participants, and none of them had any previous experience of participating in an IPE program. The answers and videos taken of the teams' performances show their enthusiasm and acceptance to be in interprofessional teams. The following are the main themes with the related quotes.

### Professional mutual recognition

Most of the textual data and observations showed that CMIPE provided a unique opportunity for the participants to interact, and learn from and about each other matters such as professional roles, responsibilities, capabilities, and limitations, which are necessary for interprofessional collaboration in crisis management and such things will not be achieved unless people have the opportunity to interact purposefully. 

As a nurse replied: *“It was an occasion for knowing each other. Unfortunately, in daily workflow, there isn’t any opportunity for us to interact and have mutual understanding, and when a crisis suddenly arises, we have to solve a problem together without having mutual understanding and shared knowledge and this is so dangerous….. Oh... problems start here (p12)*.

Ambiguity in professional roles is a common problem in critical situations, especially in unpredictable crises. The participants' experiences showed that they understood the importance of mutual recognition and clarification of roles in critical situations. Another nurse had written:

*“One of our biggest challenges in critical situations is ambiguity or confusion in roles. These programs help us to clarify different roles in critical situations”* (p14).

The participants' comments also showed CMIPE was an opportunity to correct misconceptions about other professions. As a nurse mentioned;

*“Sometimes in my interactions with others, I am wrong; I noticed some of my perceptual mistakes. Some of my perceptions were wrong. And this was an opportunity for me to correct some of my mistakes”* (p23).

### Shared mental models

The most important negative consequence of separated education in dealing with a crisis is the lack of a common vision and perception among professional groups involved in the crisis. The shared mental model has been known as a critical requirement of teamwork. Problem- solving in the interprofessional teams needs a common image of different phenomena or situations. Discussion and reflection as well as critical thinking are the most significant steps to reach common perceptions among members of the team. 

A nurse said: *“Despite the different opinions and knowledge of each of us with respect to our professional role, in order to solve the problem in the presented scenarios, we had to share our views and come up with an agreed conclusion”* (p13).

Another nurse had written; *“It would not be possible to solve critical scenarios unless we reached a common understanding of the issue, and this is achieved with the interaction and dialogue by the team members”* (p16).

Another nurse said: *“It didn’t matter how different or contradictory we thought, but what mattered was that we finally came up with a common plan. We argued so much that we finally understood each other”* (p7, p15).

### Valuing joint responsibility and collaboration

Joint responsibility, common goal setting, and shared decision-making are the most important principles of teamwork and crisis management which were perceived by the participants. However, they stressed that these factors have been neglected in crisis management in Iran.

As a nurse said; *“I have participated in many maneuvers, but in them, the interaction between professions was not seen at all…, but at this maneuver, it was very amazing for me that people of different professions sit together and exchange their ideas and information. In previous maneuvers, each one was just…, we focused on our work, not on relationships and collaborations that could strengthen and move the work forward”* (p11).

A nurse had written; *“My experience of being at the Varzeqan earthquake (northwestern Iran) in August 2012 was very bitter and terrible; the inconsistency and lack of teamwork and clear plan among the relief forces and health professions were the important factors that increased damages. If the forces had already been trained for collaboration in these situations….oh....perhaps many people would have been alive now”* (p14).

A nurse replied; *“We had to make a decisive decision together. Resolving scenarios in the team and dealing with them required coordination and responsibility of all team members. When difficult situations like crises occur, all your presumptions may disconfirm and you have to make quick decisions and act quickly; this requires a prior preparation, coordination, and collective responsibility; otherwise, you will lose everything”* (p22).

### Perceived self-worth as a member of an interprofessional team

Interprofessional maneuvering was a great opportunity to see and listen to the nurses and become aware of their abilities. In our context, the current workflow indicates a great emphasis on the hierarchy and levels of power in interprofessional interactions, which is associated with ignoring the roles and views of nurses and as a result, decreasing nurses’ self-confidence and self-worth. Physicians are often at the top of the organizational hierarchy and underestimate nurses' roles. This is the most well-known destructive factor in the collaborative relationship between physicians and nurses in our context. In this study, nurses experienced a sense of trust and acceptance. Self-perception as a member of an interprofessional team is an important step towards gaining an interprofessional identity. 

As a nurse stated: *“My good experience in the group was mutual acceptance and respect so that I could easily express my views without worrying about ridicule or being judged”* (p20).

Mainly in interprofessional teams, nurses, as the leader and representative, presented the team report and this shows the importance and attention to the position of military nurses in critical situations. 

A nurse had written: *“It was a wonderful experience for me. I felt proud to be a member of an interprofessional team and to have the opportunity to show my abilities to others and my abilities were interesting to them”* (p3).

Another nurse said: *“It was an honor for me when a firefighter asked me what I could do for a patient with cardiac arrest before the doctor arrives and when I explained to him, he listened eagerly and took notes”* (p17).

## Discussion

This study aimed to explore military nurses’ experiences of participating in crisis management education based on an interprofessional approach. Professional mutual recognition, shared mental models, valuing joint responsibility, and collaboration and the perceived self-worth as a member of an interprofessional team are the emergent themes, which describe military nurses' experiences of participating in this educational program. IPE is not known enough to be used in practice in Iran. This program was conducted for the first time. It was very welcomed by the participants and the officials of the Military Nursing School. It was an important step in recognizing and applying IPE in the field of crisis management and health. 

Numerous studies are consistent with the findings of the present study. Atack, et al. in a study assessed the effectiveness of an e-learning crisis management program based on an interprofessional approach on the capabilities of the students of several military and civilian professional groups involved in crisis including medicine, nursing, paramedical, police, and media management. Their educational program included teaching the basics of crisis management, film presentation, group discussion, and participation in a crisis simulation. The findings showed enhancement of the participants' abilities to respond to the crisis, awareness of the roles of other groups involved in the crisis, and their abilities to provide teamwork in a crisis situation ( 34 ). Miller, et al. designed and implemented an interprofessional course in crisis management for 312 students from different health sciences. The findings showed that the presence of students in a 10-hour IPE program and simulation can effectively improve their knowledge, attitude, and practice to prepare and respond to critical situations and interprofessional collaborations ( 35 ). Livingston, et al. designed a broad IPE program to develop teamwork skills in critical situations for nursing students. This program called "disaster day", included 35 nursing students and their faculty, along with 300 students from seven disciplines involved in the crisis and 500 volunteers from various military and civilian organizations. The program was the simulation of the events of a critical day with high casualties that students managed it with interprofessional collaboration. The simulated program helped the participants to increase their abilities of teamwork, critical thinking skills, and decision making in crises. One of the strengths of this program is the presence of 500 volunteers, thus increasing public awareness of interprofessional collaboration in critical events ( 36 ). Kim, et al. also designed a simulated e-learning IPE to deal with the crisis. Four hundred and two students from four professions of health sciences including medicine and nursing participated in the program. Measurements before and after the intervention showed an increase in students' abilities to respond to crises, their collaboration and teamwork ( 37 ). Digregorio, et al. in a simulated IPE to deal with the crisis measured nursing students' perceptions of interprofessional collaboration, mutual roles recognition, conflict management and client-centeredness before and after education. Their findings indicated the cognitive and functional weakness of students in teamwork and recommended the need to integrate interprofessional competencies in crisis management educational programs ( 28 ). In one study, senior nursing students' perceptions of an interprofessional simulation-based education were examined. Four major themes expressed nurses' perceptions, including understanding roles and responsibilities, enhancing collaboration, improving personal and interpersonal skills, as well as patient outcomes ( 38 ). Another study examined nursing students' experiences of participating in an interprofessional education program and indicated that interprofessional communication, mutual understanding of roles, responsibilities of the healthcare team, and knowledge of interprofessional collaborative practice are the most practical experiences of students ( 39 ). In fact IPE, by placing people from different professions in an effective interaction, provides an opportunity to learn from each other, together and about each other, thus improving collaboration, mutual understanding, trust, shared collective responsibility, and shared decision making among health professions ( 20 , 21 ). In a study examining the experiences of nursing and other health students attending a simulated interprofessional education program in the intensive care unit, three key themes were discovered, including interprofessional teamwork, knowing roles and responsibilities, as well as increased confidence in treatment skills. The authors stated the simulated interprofessional education experiences may provide an opportunity for students and institutions to collaborate and provide additional engagement with healthcare professions that may not be presented within a single institution ( 40 ). Approximately most of the studies in this field confirm our results. In reviewing the relevant literature, no findings contradicted the findings of the present study, and this indicates the effectiveness of team-based and interprofessional educational interventions.

### Limitations

One of the limitations of this study was the difficulty of coordinating for the presence of guests from other organizations such as fire and rescue services, and Red Crescent. Another problem was the military nurses and doctors’ attending in a two-day program, as the number of staff is small and their workload is high in military hospitals. The participation of military nurses in the study was done through recall and was completely voluntary, which can affect the findings. Also, similar to all qualitative studies, the generalizability of the findings is not considered, hence more studies are required to generalize the findings. In fact, IPE is in its infancy and more studies are needed to develop and apply it. Another issue to be considered in this study is that our participants were Persian-speaking and their comments and experiences were translated; one translator translated their words into English and another translator translated the English text into Persian and adapted it to the original text. In addition, socio-cultural and perceptual differences must be taken into account.

## Conclusion

The crisis management education based on an interprofessional approach created new experiences for military nurses by bringing together professional groups involved in a crisis in a constructive interaction, and with emphasis on learning from each other. This empowers them to provide teamwork and interprofessional collaboration in a critical situation, and therefore, enhancing their ability to cope with different crises. Nowadays, with the spread of various crises in the world, moving towards interprofessional education in crisis management is not a choice, but a necessity. It can be used in the educational planning of medical and military organizations.
